# Supplementary material for: Paired yeast one-hybrid assays to detect DNA-binding cooperativity and antagonism across transcription factors
Source: Nat Commun. 2023 Oct 18;14:6570. doi: 10.1038/s41467-023-42445-6 (PMC10584920; doi:10.1038/s41467-023-42445-6)
Supplement: Supplementary file 1 — Supplementary Information [file 41467_2023_42445_MOESM1_ESM.pdf]

Supplementary information

Paired yeast one-hybrid assays to detect DNA-binding cooperativity and antagonism across transcription factors

Anna Berenson[1], Ryan Lane[1], Luis F. Soto-Ugaldi[2], Mahir Patel[3], Cosmin Ciausu[3], Zhaorong Li[1], Yilin Chen[1], Sakshi Shah[1], Clarissa Santoso[1], Xing Liu[1], Kerstin Spirohn[4,5,6], Tong Hao[4,5,6], David E. Hill[4,5,6], Marc Vidal[4,5,6], Juan I. Fuxman Bass[1,4*]

[1]Department of Biology, Boston University, Boston, MA 02215

[2]Tri-Institutional Program in Computational Biology and Medicine, New York, New York

[3]Department of Computer Science, Boston University, Boston, MA 02215

[4]Center for Cancer Systems Biology (CCSB), Dana-Farber Cancer Institute, Boston, MA, 02215

[5]Department of Cancer Biology, Dana-Farber Cancer Institute, Boston, MA, 02215

[6]Department of Genetics, Blavatnik Institute, Harvard Medical School, Boston, MA 02115

* Correspondence to: J.I.F.B. (fuxman@bu.edu).

Supplementary Figures

Supplementary Figure 1: Reproducibility of pY1H assays

Supplementary Figure 2: pY1H experimental and analysis pipeline

Supplementary Figure 3: Features of DISHA

Supplementary Figure 4: Cooperative and antagonistic relationships detected by pY1H assays

Supplementary Figure 5: TF family representation

Supplementary Figure 6: Cooperative and antagonistic events from pY1H assays

Supplementary Figure 7: Overlap between pY1H data and motif predictions

Supplementary Figure 8: DNA binding specificity for TF-pairs

Supplementary Figure 9: Predicted structures of STAT1-STAT3 dimers

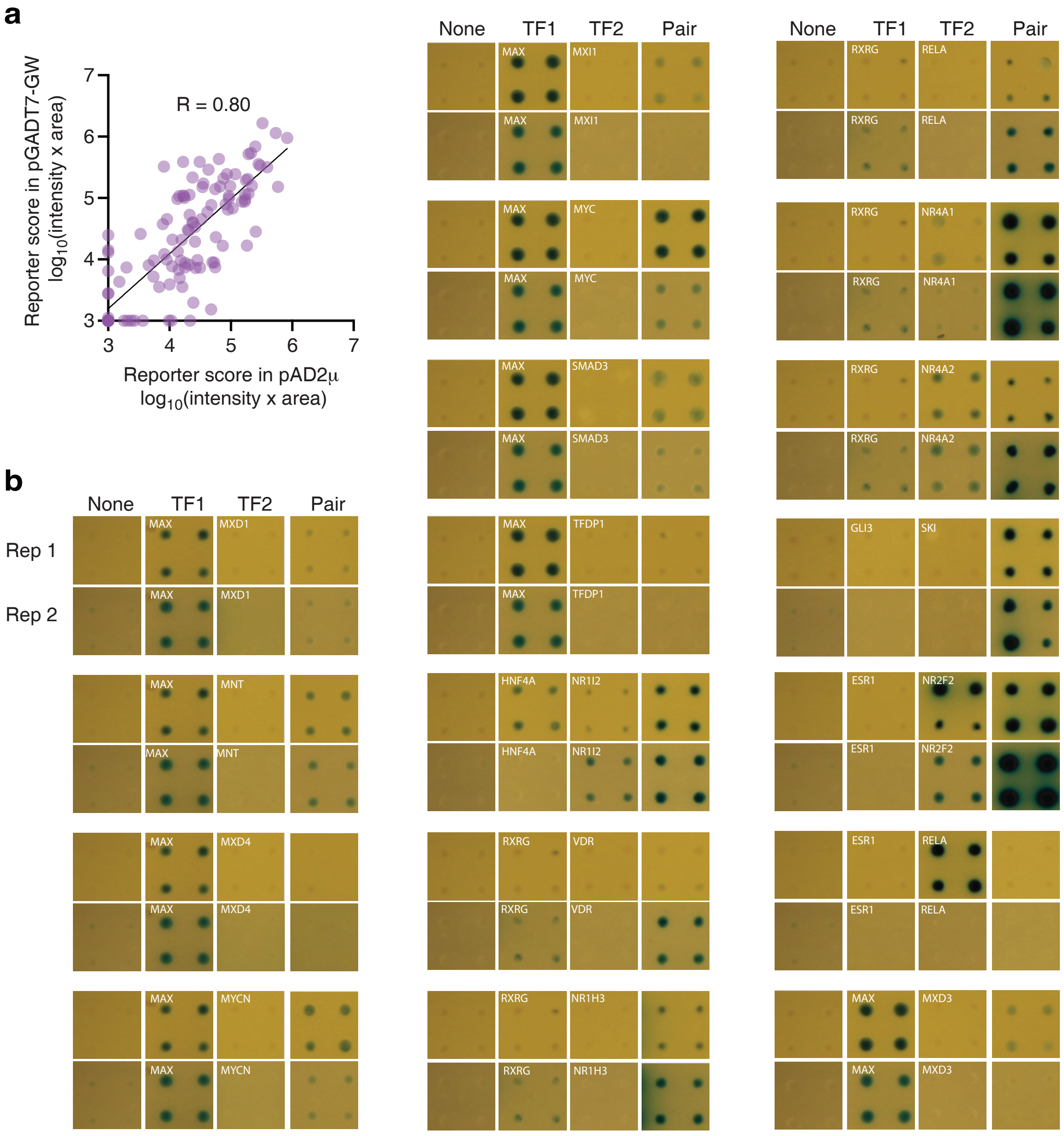

Supplementary Figure 1. Reproducibility of pY1H assays. (**a**) Correlation between reporter scores (intensity x area) for single-TFs (n = 120) cloned into the pGADT7-GW and the pAD2μ vectors and transformed into yeast. Correlation by Pearson Correlation Coefficient. (**b**) Cooperative and antagonistic interactions from two pY1H screens using the 2-AD design conducted on different days for the *CCL15* promoter. Source data are provided as a Source Data file.

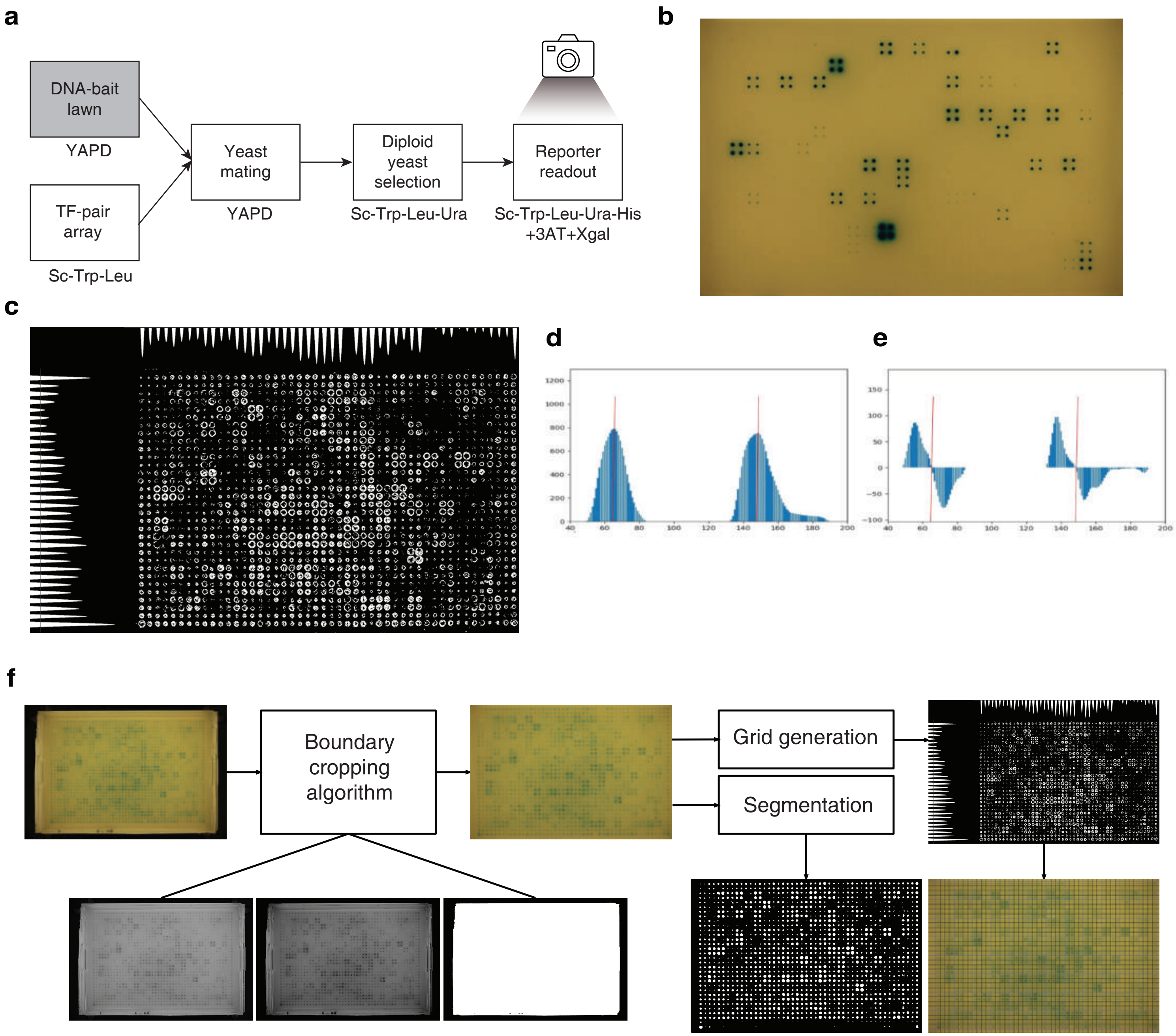

Supplementary Figure 2. pY1H experimental and analysis pipeline. (**a**) Experimental pipeline for pY1H assays. A lawn of the yeast DNA-bait is mated with the TF-pair array in a YAPD plate for 1 day. Diploid yeast are selected in Sc-Trp-Leu-Ura plates for 2 days and then transfered to readout plates containing 3AT and X-gal. Pictures are taken at days 2, 3, 4 and 7. (**b**) Example of a pY1H readout plate. (**c**) Vertical and horizontal histogram projection of a sub-optimal segmentation mask. Each peak corresponds to the center of the colony. (**d**) Plot of a 160-pixel region of histogram projection. (**e**) Plot of the first-order derivative of the region of the plot in d. Each "zero-crossing" event corresponds to the histogram's peak or the colony's center. (**f**) Pipeline of the image analysis process. First, the boundary cropping algorithm selects the region of interest. Then the grid generation algorithm performs histogram projection analysis to generate a grid based on the TF-pair array coordinates. Finally, the segmentation model generates a segmentation map for the colonies and determine colony area and intensity of blue color.

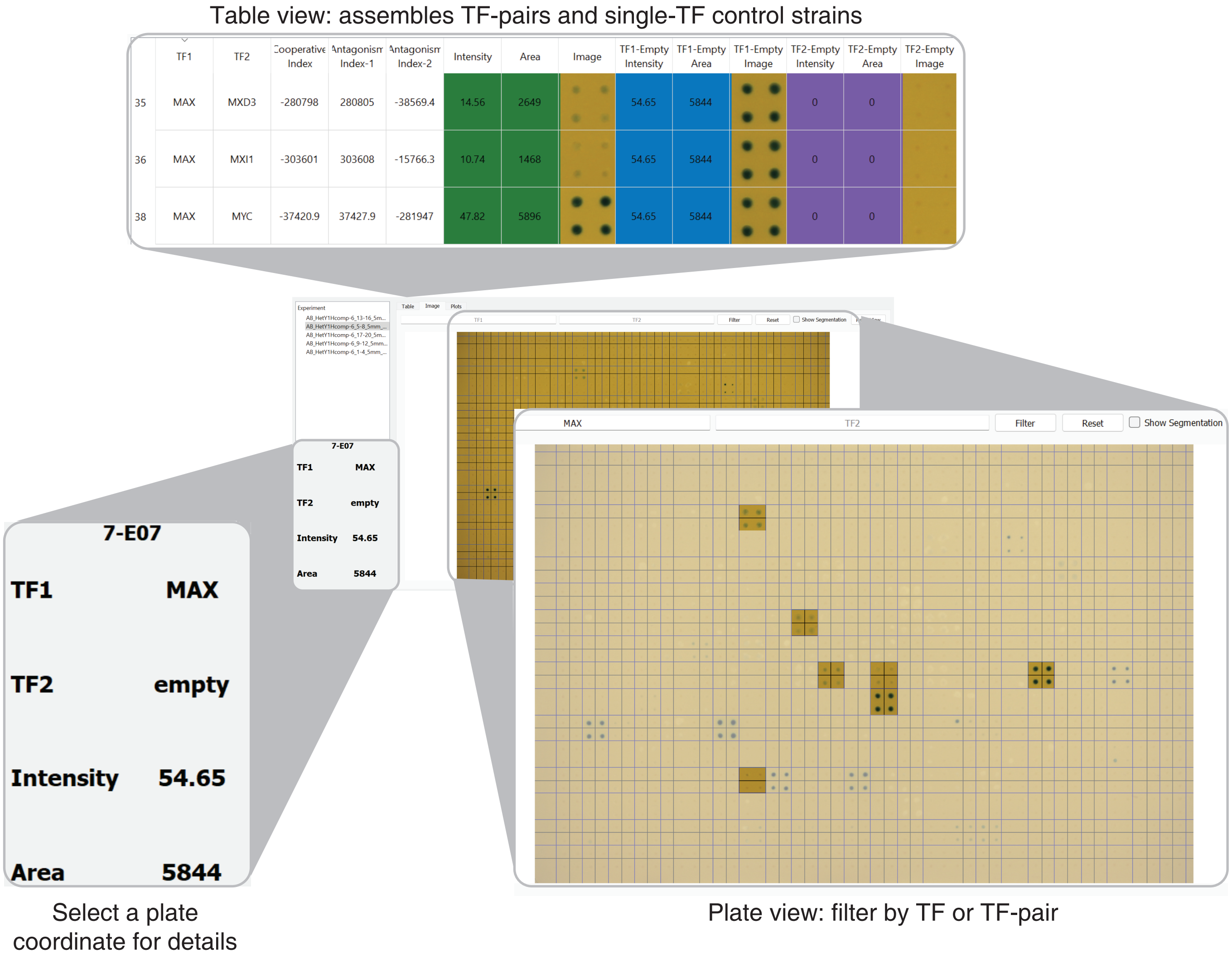

Supplementary Figure 3. Features of DISHA. The Plate view displays plate images derived from pY1H assays and allows to filter by singe-TF or TF-pair. Users can also select an array coordinate to identify the TF-pair, reporter intensity measured as blue color, and area of colonies. The Table view displays images of TF-pair colonies alongside single-TF colonies allowing side-by-side comparisons, together with reporter intensity and colony area. In addition, the table presents the cooperative and antagonistic indices calculated for each TF-pair.

a

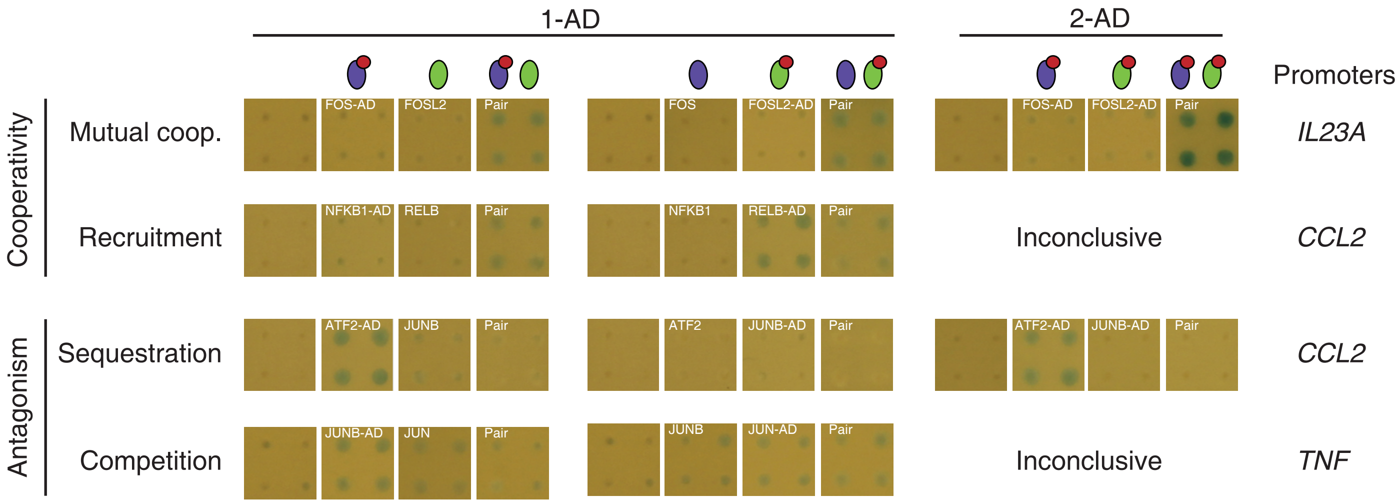

b

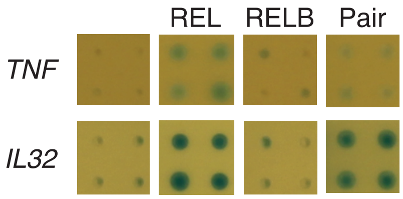

Supplementary Figure 4. Cooperative and antagonistic relationships detected by pY1H assays. (**a**) Different types of cooperative (mutual cooperativity and recruitment) and antagonistic (sequestration and competition) events detected using the reciprocal 1-AD and the 2-AD pY1H designs. Cases that cannot be determined using the 2-AD design are indicated as inconclusive. (**b**) Examples of REL-RELB pairs tested against the *TNF* and *IL32* promoters using the 2-AD design.

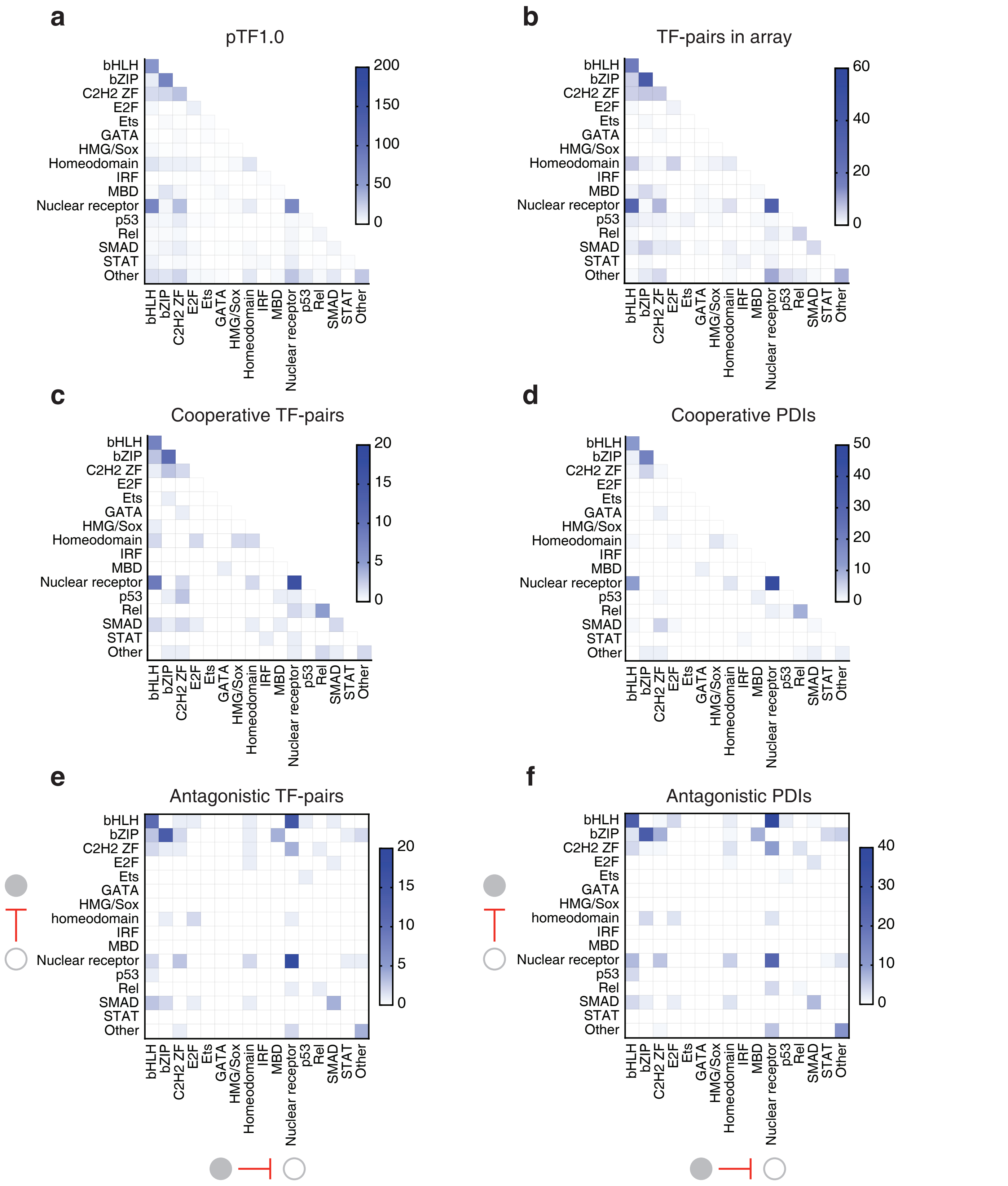

Supplementary Figure 5. TF family representation. (**a**, **b**) Number of TF-pairs for each TF family combination for the 868 TF-pairs in pTF1.0 (**a**) and the 297 sequence-confirmed TF-pairs in the array (**b**) (as in Fig. 3b,c). (**c, d**) Number of cooperative TF-pairs (**c**) and PDIs (**d**) identified in a pY1H assay against 18 cytokine promoters. (**e, f**) Number of antagonistic TF-pairs (**e**) and PDIs (**f**) identified in a pY1H assay against 18 cytokine promoters. Columns indicate the family of the antagonistic TF and rows indicate the family of the antagonized TF. Source data are provided as a Source Data file.

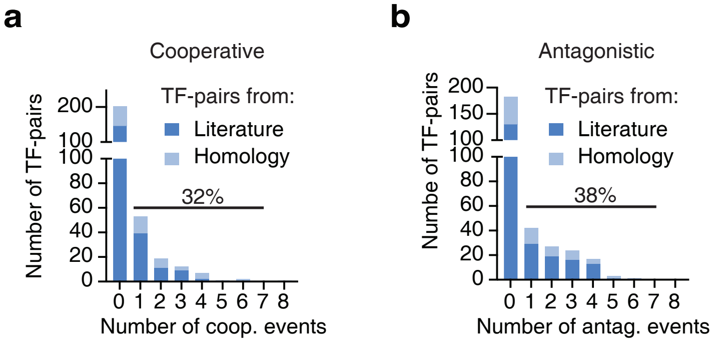

Supplementary Figure 6. Cooperative and antagonistic events from pY1H assays. Number of TF-pairs involved in different numbers of cooperative (**a**) and antagonistic (***b***) events for TF-pairs derived from the literature or predicted based on homology. The percentage of TF-pairs with at least one cooperative or antagonistic event is indicated. Source data are provided as a Source Data file.

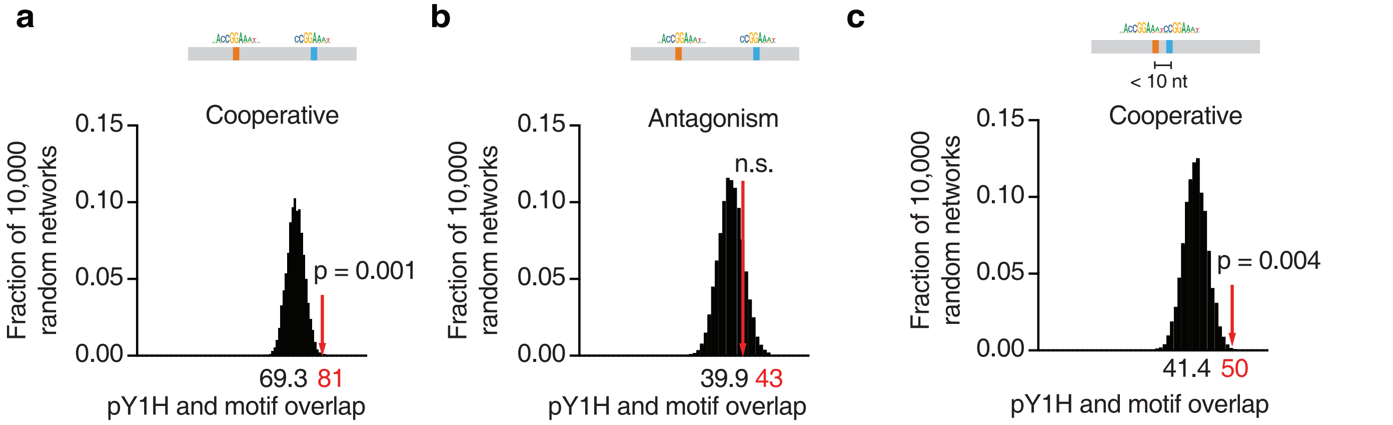

Supplementary Figure 7. Overlap between pY1H data and motif predictions. (**a, b**) Overlap between pY1H cooperative (**a**) and antagonistic (**b**) events with the presence of motifs for both single-TFs in the corresponding promoter sequences. The histograms show the distribution of the overlap with each randomized pY1H network, where the pY1H network was randomized 10,000 times by edge-switching (each edge is a TF-pair and a cytokine promoter). The numbers under the histograms indicate the average overlap in 10,000 randomized networks, while the red arrows indicate the observed overlap with the actual pY1H-derived network. Two-tailed statistical significance was calculated from Z-score values assuming normal distribution for overlap with the randomized networks. (**c**) Overlap between pY1H cooperative events with the presence of motifs for both single-TFs within 10 bp in the corresponding promoter sequences. Source data are provided as a Source Data file.

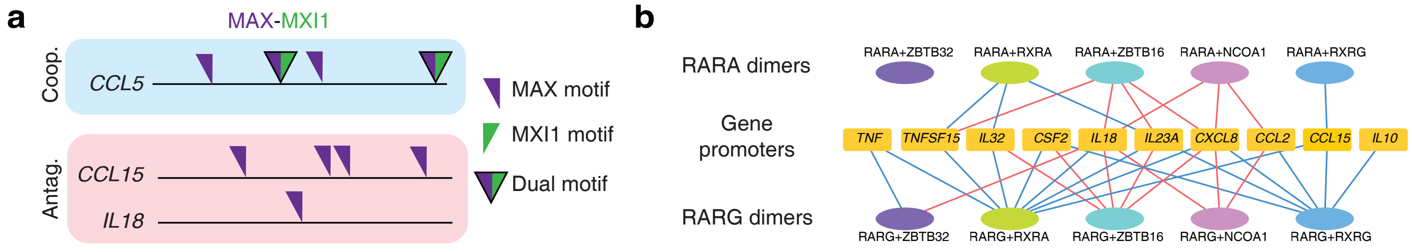

Supplementary Figure 8. DNA binding specificity for TF-pairs. (**a**) Distinct and overlapping motifs for MAX and MXI1 in the indicated cytokine gene promoters. (**b**) Cooperative and antagonistic events for TF-pairs involving paralogs RARA and RARG. Source data are provided as a Source Data file.

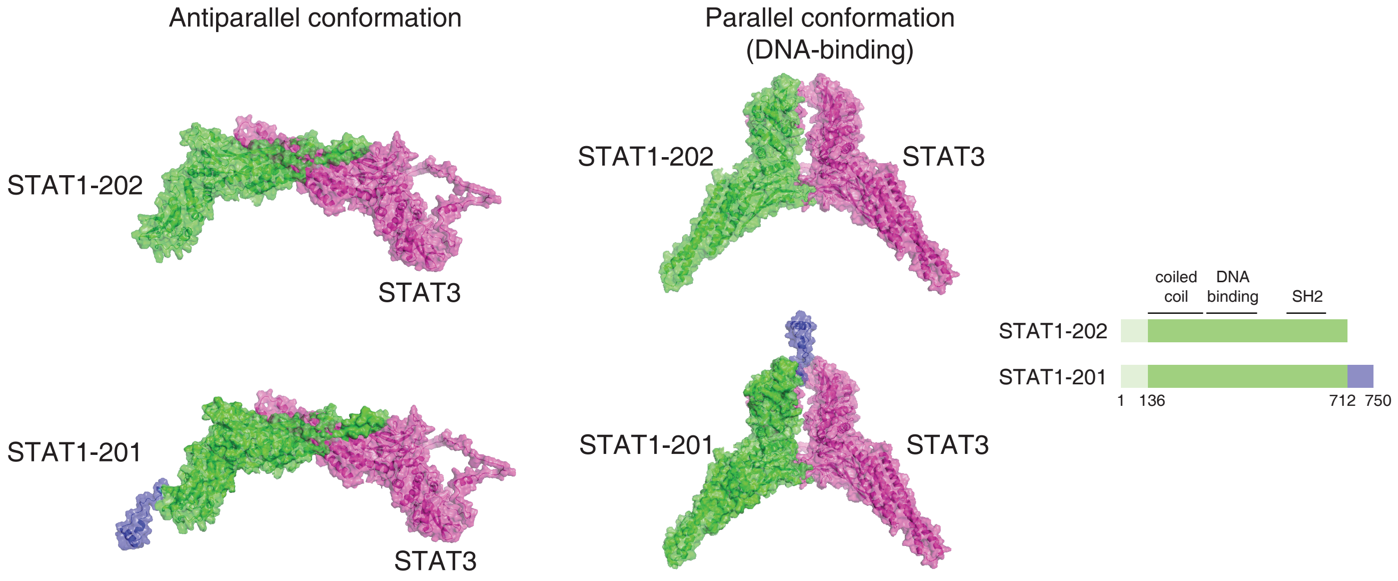

Supplementary Figure 9. Predicted structures of STAT1-STAT3 dimers. Structures of STAT1 isoforms and reference STAT3 isoform predicted using Alphafold 2 were arranged in the antiparallel conformation (unbound to DNA) or parallel conformation (adopted for DNA binding). Amino acids 136-712 of STAT1 are colored in green, the additional sequence (713-750) of STAT1-201 is colored in blue, and STAT3 is colored in magenta.
