# Supplementary material for: Paired yeast one-hybrid assays to detect DNA-binding cooperativity and antagonism across transcription factors
Source: Nat Commun. 2023 Oct 18;14:6570. doi: 10.1038/s41467-023-42445-6 (PMC10584920; doi:10.1038/s41467-023-42445-6)
Supplement: Supplementary file 3 — Reporting Summary [file 41467_2023_42445_MOESM3_ESM.pdf]

nature portfolio

Corresponding author(s): Juan Fuxman Bass

Last updated by author(s): Sep 11, 2023

Reporting Summary

Nature Portfolio wishes to improve the reproducibility of the work that we publish. This form provides structure for consistency and transparency in reporting. For further information on Nature Portfolio policies, see our Editorial Policies and the Editorial Policy Checklist.

Statistics

For all statistical analyses, confirm that the following items are present in the figure legend, table legend, main text, or Methods section.

n/a	Confirmed	
☐	☒	The exact sample size (*n*) for each experimental group/condition, given as a discrete number and unit of measurement
☒	☐	A statement on whether measurements were taken from distinct samples or whether the same sample was measured repeatedly
☐	☒	The statistical test(s) used AND whether they are one- or two-sided<br>*Only common tests should be described solely by name; describe more complex techniques in the Methods section.*
☒	☐	A description of all covariates tested
☐	☒	A description of any assumptions or corrections, such as tests of normality and adjustment for multiple comparisons
☐	☒	A full description of the statistical parameters including central tendency (e.g. means) or other basic estimates (e.g. regression coefficient) AND variation (e.g. standard deviation) or associated estimates of uncertainty (e.g. confidence intervals)
☐	☒	For null hypothesis testing, the test statistic (e.g. *F*, *t*, *r*) with confidence intervals, effect sizes, degrees of freedom and *P* value noted<br>*Give P values as exact values whenever suitable.*
☒	☐	For Bayesian analysis, information on the choice of priors and Markov chain Monte Carlo settings
☒	☐	For hierarchical and complex designs, identification of the appropriate level for tests and full reporting of outcomes
☒	☐	Estimates of effect sizes (e.g. Cohen's *d*, Pearson's *r*), indicating how they were calculated

Our web collection on statistics for biologists contains articles on many of the points above.

Software and code

Policy information about availability of computer code

Data collection	We developed a pipeline for processing and visualizing yeast plate images: Detection of Interactions Software for High-Throughput Analyses (DISHA). Code is available at https://github.com/jfuxman/PY1H_NatComm2023. Additional information can be found in the manuscript Methods.
Data analysis	Next-generation sequencing data were analyzed using the following tools: Fast QC v.0.11, MultiQC, cutadapt 4.1, the BIOMART R package, Samtools 1.10, and an in-house R script primarily based on Rsamtools functions. Motif analysis was conducted using the TFMPvalue R package. Network randomization was performed using the igraph R package. Paralog partner similarity was determined using the seqinr R package. Expression analysis was performed using Harmony and the Seurat R package. Structure predictions were performed using ALphafold 2 and Pymol. Statistical analyses were performed using GraphPad Prism version 9. Additional information can be found in the manuscript Methods.

For manuscripts utilizing custom algorithms or software that are central to the research but not yet described in published literature, software must be made available to editors and reviewers. We strongly encourage code deposition in a community repository (e.g. GitHub). See the Nature Portfolio guidelines for submitting code & software for further information.

Data

Policy information about availability of data

All manuscripts must include a data availability statement. This statement should provide the following information, where applicable:

- Accession codes, unique identifiers, or web links for publicly available datasets
- A description of any restrictions on data availability
- For clinical datasets or third party data, please ensure that the statement adheres to our policy

All data generated during this study are included in this published article and its Supplementary Information/Source Data files. Sequencing data can be found at the NCBI Sequence Read Archive at accession number PRJNA1015222 (https://www.ncbi.nlm.nih.gov/bioproject/PRJNA1015222) . Yeast plate images are available at https://doi.org/10.7910/DVN/GITY2H. Enhanced yeast one-hybrid data can be found at https://doi.org/10.1093/nar/gkaa1055. The CytReg database can be found at https://cytreg.bu.edu/search_v2.html. ChIP-seq data were obtained from the GTRD database (https://doi.org/10.1093/nar/gkaa1057; http://gtrd.biouml.org/). DNA binding motif data were obtained from the CIS-BP database (https://doi.org/10.1016/j.cell.2014.08.009; http://cisbp.ccbr.utoronto.ca/). Expression data were obtained from the Tabula Sapiens atlas (https://doi.org/10.1126/science.abl4896). All clones and yeast strains generated in this study are available upon request made to corresponding author J.I.F.B., and will be shipped within one month of request.

Research involving human participants, their data, or biological material

Policy information about studies with human participants or human data. See also policy information about sex, gender (identity/presentation), and sexual orientation and race, ethnicity and racism.

Reporting on sex and gender	N/A
Reporting on race, ethnicity, or other socially relevant groupings	N/A
Population characteristics	N/A
Recruitment	N/A
Ethics oversight	N/A

Note that full information on the approval of the study protocol must also be provided in the manuscript.

Field-specific reporting

Please select the one below that is the best fit for your research. If you are not sure, read the appropriate sections before making your selection.

☒ Life sciences ☐ Behavioural & social sciences ☐ Ecological, evolutionary & environmental sciences

For a reference copy of the document with all sections, see nature.com/documents/nr-reporting-summary-flat.pdf

Life sciences study design

All studies must disclose on these points even when the disclosure is negative.

Sample size	No statistical method was used to predetermine sample size. The number of DNA regions of interest selected for screening was based on feasibility considerations gleaned from previous experiments. When generating protein-pair arrays, we started with all protein-pairs known or suspected to interact with one another, and we report data corresponding to all pairs for which yeast strains were sequence-confirmed.
Data exclusions	As established prior to data collection, data were excluded for a protein-pair if the protein-pair or either corresponding single-protein yeast strain were deemed “inconclusive” by one or more of the following criteria: yeast strain was not sequence-verified; yeast strain did not show adequate growth in the array; yeast strain did not display at least 3 uniform colonies; yeast strain was contaminated during screening.
Replication	As demonstrated in our Supplementary Information, we conducted two replicate screens for the CCL15 promoter and observed a high level of reproducibility in event calling. Replicate screens of other promoters were also successful. All interactions are tested in quadruplicate colonies and we require uniform reporter signal from 3 out of 4 replicate colonies for an interaction to be considered.
Randomization	We randomized locations of yeast strains in our array plates so that strains expressing a given protein were dispersed throughout the plate. Similarly, we distributed “empty” control yeast strains, which were used for normalization, throughout each array plate to avoid any biases that might arise based on plate location.
Blinding	As no group allocations were involved in this study, researcher blinding was not applicable. Although researchers were not blinded to the identity of yeast strains during analysis, unbiased results were ensured as follows. First, yeast strains were sorted according to objective cooperativity and antagonism indexes prior to manual curation to generate an initial event list. Second, researchers used an unlabeled full plate layout view to blindly identify “positive” colonies. The initial list of cooperative and antagonistic events was then further curated to include only those that involved blindly selected “positive” colonies.

Reporting for specific materials, systems and methods

We require information from authors about some types of materials, experimental systems and methods used in many studies. Here, indicate whether each material, system or method listed is relevant to your study. If you are not sure if a list item applies to your research, read the appropriate section before selecting a response.

Materials & experimental systems

n/a	Involved in the study
☒	☐ Antibodies
☒	☐ Eukaryotic cell lines
☒	☐ Palaeontology and archaeology
☒	☐ Animals and other organisms
☒	☐ Clinical data
☒	☐ Dual use research of concern
☒	☐ Plants

Methods

n/a	Involved in the study
☒	☐ ChIP-seq
☒	☐ Flow cytometry
☒	☐ MRI-based neuroimaging
