# Supplementary material for: Paired yeast one-hybrid assays to detect DNA-binding cooperativity and antagonism across transcription factors
Source: Nat Commun. 2023 Oct 18;14:6570. doi: 10.1038/s41467-023-42445-6 (PMC10584920; doi:10.1038/s41467-023-42445-6)
Supplement: Supplementary file 4 — Description of Additional Supplementary Files [file 41467_2023_42445_MOESM4_ESM.pdf]

Description of Additional Supplementary Files

Supplementary Data File
Description: This file contains Supplementary Tables 1-16, as well as detailed notes about each table. These include information about DNA baits, protein ORF clones, protein pairs studied, pY1H events, and TF expression data.
